# Prospective Evaluation of Cardiovascular, Cardiorespiratory, and Metabolic Risk of German Office Workers in Comparison to International Data

**DOI:** 10.3390/ijerph17051590

**Published:** 2020-03-01

**Authors:** Markus Strauss, Peter Foshag, Roman Leischik

**Affiliations:** 1 Department of Cardiology, Sector Preventive Medicine, Health Promotion, Faculty of Health, School of Medicine, University Witten/Herdecke, 58095 Hagen, Germany; 2 Department of Cardiology I—Coronary and Peripheral Vascular Disease, Heart Failure Medicine, University Hospital Muenster, Cardiol, 48149 Muenster, Germany

**Keywords:** office workers, physical fitness, risk factor, cardiovascular risk, metabolic risk

## Abstract

Background: Employment in the administrative sector is characterized by prolonged sedentary work, which has been tied to increased morbidity and compromised health. The aim of this study was to determine cardiovascular, cardiorespiratory and metabolic risk parameters of German office workers (OWs) in comparison to OWs from other nations. Material and Methods: A total of 46 male office workers from the North Rhine-Westphalia region (Germany) participated in the survey. Anthropometric measurements, cardiovascular and metabolic risk factors, as well as laboratory parameters were taken. The 10-year cardiovascular risk was calculated by using the Framingham risk score. The diagnosis of metabolic syndrome was based on the criteria of the International Diabetes Federation. Cardiorespiratory status was assessed by exercise spirometry. Results: The analyzed group of OWs demonstrated a high prevalence of preobesity (Body Mass Index 26.4 ± 4 and waist circumference 97.3 ± 11.7 cm) and 58.7% of the OWs showed an abnormally large waist circumference. Cardiovascular risk was correspondingly elevated as compared with other international studies (9.7% ± 9.2%). High risk cardiovascular profiles were detected in 10.7% of the participants and 33% of the OWs in our study group were diagnosed with metabolic syndrome. The oxygen uptake of the OWs was 34.1 ± 8.1 mL/kg^−1^·min^−1^. Conclusions: The German OWs show elevated cardiovascular risk assessed using the Framingham risk score and also a high tendency for metabolic syndrome. The OWs need to be made further aware of the cardiovascular risk and resulting health implications. Implementation of health promotion concepts such as corporate sports activities or nutrition courses should be taken into consideration to counteract cardiovascular risk factors and the subsequent development of cardiovascular disease in later life.

## 1. Introduction

Physical activity is recognized as one of the most important factors protecting against cardiovascular disease and cancer worldwide [1,2,3].

Healy et al. [4] described harmful associations of prolonged sedentary time with cardiometabolic and inflammatory biomarkers in U.S. adults. In the 1950s, research showed that employment requiring high physical activity at work had lower rates of cardiovascular-associated diseases as compared with physically inactive work [5]. Morris et al. were the first to describe the correlation between physical activity and a lower incidence of ischaemic heart disease.

The sector of public administration in Germany is a numerically large workforce of approximately 18.5 million German citizens [6]. It is known that civil servants show different health behaviors with an associated poor prognosis of health as compared with the general population [7]. Working as an office worker (OW) in an administrative authority is characterized as sedentary work. These work-related sedentary activities are associated with increased cardiovascular risk [4]. Similarly, studies that have analyzed data from different countries have shown that across countries OWs are more likely to lead an unhealthy lifestyle and exhibit physical inactivity [8]. In large international cohorts, physical inactivity could be held responsible for up to 25% of breast and colon cancers, up to 27% of the diabetes mellitus burden, and 30% of cases of coronary heart disease worldwide [9].

Studies investigating German OWs are rare, although it is of major medical and socioeconomic interest to improve the prevention of work-related development of disease. This study prospectively examined cardiovascular, cardiorespiratory, and metabolic parameters in German OWs in order to collect epidemiographic risk parameters associated with this occupational group. After conducting the study, a literature search was performed to compare our findings to international data. Our aim was to evaluate differences in cardiovascular and metabolic risks amongst countries.

## 2. Materials and Methods

### 2.1. Participants

Male sedentary OWs in the Ruhr area (Dortmund, Hagen, and Witten) were invited to participate in this study via internet advertisements, social media, and local corporate distribution after they responded to an official request. The recruitment of study participants was advertised at workplaces. Participation was voluntary. The inclusion criterion was employment in administrative offices with a predominantly sedentary occupation. A group of 46 participants was included. The group consisted of male Caucasians ranging in age from 26 to 62 years. The OWs were civil servants who worked in tax offices or municipal administration and performed desk work. Desk work was characterized by a sedentary position of work in a fulltime job (>35 hours per week) in accordance with the criteria of Sedentary Behavior Research Network (SBRN) [10]. The participants belonged to the same social stratum (“middle class”).

Examinations were performed at baseline and at one time at the Sports Medicine Centre in Hagen (Research Sector Prevention, Public Health and Sports Medicine, University Witten/Herdecke) by a trained clinician.

### 2.2. Assessment Tools and Procedures

A questionnaire was used to collect information which included individual sedentary time at work (hours) and use of tobacco and alcohol, as well as to calculate the metabolic equivalents (METS) based on Ainsworth et al. [11]. Values were assessed for one week. Dynamic sport activities such as jogging, cycling, swimming, football, martial arts, and anaerobic exercise were summarized under the term dynamic METS and one MET corresponded to 1 kcal·kg^−1^·h^−1^.

The measurement of blood pressure was performed in a supine position with calibrated standard blood pressure cuffs. For the classification of blood pressure, the 2018 ESH/ESC guidelines for management of hypertension were used (systolic blood pressure: ≥140 mmHg hypertension Grade I, ≥160 mmHg Grade II, and ≥180 mmHg Grade III) [12]. Resting heart rate was measured by a 12-channel electrocardiogram ECG (MAC 600, General Electric Healthcare GmbH, Solingen, Germany). Waist circumference was measured at the end of expiration in a standing position in the center of the lower edge of the ribs and the upper edge of the iliac crest.

Participants were asked to fast (refrain from eating and drinking for 4 to 6 hours prior to the examination) before measuring body weight and having blood examinations in the morning. Body weight and body composition were determined using the body composition analyser BC-418MA (Tanita UK Ltd., The Barn, Philpots Close, Yiewsley, West Drayton, Middlesex, UB7 7RY, UK) [13]. For the measurements of body weight, body composition, and body height, participants were instructed to wear only comfortable shorts with no other clothing or shoes. Blood serum parameters that were analyzed included: total cholesterol, high-density lipoprotein (HDL), low-density lipoprotein (LDL), triglyceride, glycated haemoglobin (HbA1c), C-reactive protein (CRP), and lipoprotein (a). Lipoprotein (a) was measured by our laboratory (Immun—Essay La Roche).

The Framingham risk score was used to calculate the cardiovascular risk scores based on observations and data from the Framingham Heart Study [14]. Specifically, in our investigations we applied the 10-year cardiovascular disease risk score calculator based on the Framingham study [15]. The following parameters were measured to calculate cardiovascular risk: age, diabetes, smoking, treated and untreated systolic blood pressure, total cholesterol, HDL levels, and Body Mass Index BMI.

The classification used to identify individuals with metabolic syndrome was based on the criteria of the International Diabetes Federation (IDF) in 2005 [16]. The definition focuses on four entities: obesity, dyslipidaemia, hypertension, and insulin resistance. For diagnosis, obesity must be present, as well as two other criteria. The main criterion of the IDF definition is central obesity which can be assessed by waist circumference or by BMI. The reference value of waist circumference was 94 cm.

Spiroergometry [17,18,19] was performed in the following manner: After successful gas and volume calibration, a stress test was conducted beginning at 50 watts and continuously increasing by 25 watts every 2 min (ramp test). The test ended when the subject could no longer maintain the predefined cadence of 80/min or if the subject was subjectively exhausted and there was no further increase in VO_2max_ after 20 sec. The spiroergometric analyses were conducted as previously described [18,19]. The ventilator aerobic threshold (AT) was defined as the first nonlinear increase in the ventilatory equivalent for oxygen without simultaneous increase of the ventilatory equivalent for CO_2_. The respiratory compensation point (RCP) was defined as the simultaneous nonlinear increase of both ventilatory equivalents, according to previously described recommendations [18,19].

### 2.3. Statistical Analysis

For statistical analysis, Stata/IC 13.1 for Windows (StataCorp., LLC 4905 Lakeway Drive, College Station, TX 77845, USA) was used. The anthropometric parameters, clinical characteristics, physical activity, and cardiorespiratory fitness parameters were described using the number of participants, means, standard deviations, and medians. Categorical characteristics were displayed by specifying absolute and relative frequency. Comparisons of the study data with other study results were completed by using means and standard deviations.

### 2.4. Ethical Statement

This study was approved by the Ethic Committee of the University Witten/Herdecke (121/2013). All participants were given information about the study and were asked to sign a consent form prior to their participation.

## 3. Results

### 3.1. Study Population and Basic Characteristics

In this prospective study, all 46 consecutively recruited male participants were included in the study. The participating OWs were between 26 and 62 years old. The mean age was 45.8 ± 10 years (Table 1). The OWs had 21.1 ± 10.8 years of professional experience varying between 3 and 46 years. The mean sedentary time at work of all participants was 52.2 ± 17.2 h/week. Leisure time activity in vigorous physical activities was 2212 ± 2293 METs/week. Strength training, jogging, swimming, and cycling were considered vigorous physical activities.

### 3.2. Cardiovascular Risk

#### 3.2.1. Cardiovascular Risk Factors

The OWs were slightly above the upper limit of the normal range of 25 kg/m² (BMI), thus they could be assigned to the pre-obese/overweight range. The OWs consumed an average of 1.72 cigarettes/day. In total, 10.9% of the OWs (*n* = 5) consumed nicotine and 71.7% of the OWs had a normal systolic blood pressure (RR ≤ 139 mmHg). The remaining participants (28.3%) had a raised systolic blood pressure at rest (RR > 140 mmHg). The diastolic elevated blood pressure values (RR ≥ 90 mmHg) were measured for 54.3% of all participants. One OW had the highest systolic blood pressure at rest with an RR of 170 mmHg. Representation of blood pressure as seen in our study group is listed in Table 2. The mean resting heart rate value was 70.1 ± 12.5 beats per minute.

The OWs showed raised total cholesterol values of 206.6 mg/dL (normal ≤200 mg/dL). The parameters HDL, LDL, triglyceride, HbA1c, and CRP were not elevated. In addition, 58.7% of the participants showed an abnormally large waist circumference and 13% had an increased BMI. By adapting the criteria of the IDF, systolic blood pressure changes were detected in 63% of the cases, whereas diastolic blood pressure changes appeared in 54.3% of the cases.

Cardiovascular risk factors were also taken into account for the diagnosis of metabolic syndrome according to the IDF [16]. In the group of examined OWs, a clear trend towards obesity became visible. When considering lipid profile percentages, elevated triglyceride was most frequently detected (39.1%). The major cardiovascular risk factors are listed in Table 3.

#### 3.2.2. Cardiovascular Risk Profile by Framingham

In Table 4, the 10-year risk for cardiovascular events and the heart/vascular age (years) shown are based on the Framingham risk score. When stratifying cardiovascular risk in groups of percentages, most OWs were classified as having a <5% chance of a 10-year cardiovascular event (32.6% of the OWs) and five participants were recognized to be high risk (>20% chance of a 10-year cardiovascular event) which represented 10.7% of the study group.

The examined OWs showed a mean 10-year cardiovascular system risk of 9.7% ± 9.2% and a mean heart/vascular age of 49.6 ± 15.5 years. They exceeded their actual mean age of 45.8 years by 3.8 years.

#### 3.2.3. Metabolic Syndrome

In the group of OWs, a metabolic syndrome was detected in 15 of 46 participants according to the criteria of the International Diabetes Federation (IDF). This corresponded to 32.6% (Table 5).

#### 3.2.4. Cardiorespiratory Fitness

The relative oxygen uptake (rel. VO_2max_) was 34.1 ± 8.1 mL/kg^−1^·min^−1^ (Table 6). An absolute oxygen uptake (abs. VO_2max_) of 2.85 ± 0.52 L/min^−1^ was shown. The maximum achieved wattage was 257.7 ± 46.9 W, while watt per kilogram was 3.03 ± 0.74 W/kg. Spiroergometry exercise parameters are displayed in Table 6.

## 4. Discussion

The collected data provides insight into cardiovascular health and cardiorespiratory fitness of German OWs. It would appear that German office workers are more likely to have metabolic syndrome and an abnormally large waist circumference as compared to office workers from other nations (e.g., Asia) [22].

### 4.1. Cardiovascular Risk

In general, sedentary time is reported as an independent risk factor for the development of cardiovascular diseases, diabetes mellitus, and higher rates of mortality [23,24,25,26,27,28]. Subjects with cardiovascular disease or high disease risk are more sedentary and less active than their healthy peers [29]. Sedentary activities of OWs represent up to 71% of their working day [30]. The average sedentary working hours of our study cohort was 52.2 ± 17.2 h/week. Past studies underline that sedentary work behavior can accelerate the emergence and progression of cardiovascular diseases [26]. This can also be assumed for our examined OWs. Therefore, future prospective observational studies are necessary.

In the examined OWs a systolic hypertension was observed in 63%, and diastolic hypertension was observed in 54.3%, based on criteria for metabolic syndrome of the IDF. Nevertheless, average systolic and diastolic blood pressure values revealed normal standard values.

The presence of hypertension is not only notable in our study cohort but can be observed in the entire population. It seemed that the prevalence of arterial hypertension in the group of OWs was higher as compared with the German population. Estimates show that slightly over 30% of the German population express arterial hypertensive blood pressure values [31]. In an international comparison, our study cohort showed the highest systolic and diastolic blood pressure values as compared with OWs from Thailand, Korea, Japan and Iran [22,32,33,34,35]. Approximately the same levels of systolic blood pressure were observed in Japanese office workers [34].

One of the most important cardiovascular risk factors is smoking. Our examined OWs showed the lowest prevalence of nicotine consumption (prevalence 10.9%) as compared with international values of nicotine consumption. British administrators, as well as Korean and Japanese OWs, showed a significantly higher prevalence of smoking [22,33,36].

When the Framingham risk scores are divided into categories of low, moderate, high, and very high risk, our study population could be classified in the “low risk” category, according to a 10-year chance of cardiovascular events that was <10% [37]. A direct comparison of the 10-year risk stratification of cardiovascular events in OWs with international studies could not be carried out due to a lack of comparative studies that used the same risk score.

In our group of OWs, the mean blood lipid levels that depict cardiovascular risk were increased above the upper limit of the healthy reference range, with the exception of total cholesterol displaying a mean of 206.6 ± 29.9 mg/dL. Our study cohort showed elevated total cholesterol values which was in agreement with the findings of previous research on "office workers". Dutch office workers displayed the highest cholesterol levels which were above the healthy range in an international comparison (mean total cholesterol 266 ± 50 mg/dL) [38]. Furthermore, relatively high triglyceride levels were identified in our study cohort. Triglyceride levels measured in our study seemed to exceed values reported by Hartung et al. (162 vs. 87.7 mg/dL), who also examined these parameters in German OWs [39]. A crucial reason for an increase in serum cholesterol levels, and thus the presence of increased cardiovascular risk in our cohort of OWs could be due to not only sedentary work but also insufficient physical activity in leisure time. In our population, vigorous physical activities amounted to 2212 METs/week. This finding could be limited on its own as calorie intake was not taken into consideration. Healy et al. [4] demonstrated in their study that a work-related sedentary lifestyle is associated with the presence of cardiovascular risk factors. The time of sedentary work activity was found to be related to an abnormally large waist circumference, elevated serum triglyceride, and serum CRP levels and lower HDL cholesterol levels [4].

In this study, we routinely analyzed lipoprotein (a) in our probands. Lipoprotein (a) represents an important cardiovascular risk parameter. Elevated values of this parameter are associated with an increased cardiovascular risk profile and can, in conjunction with other cardiovascular risk factors, increase the risk for vascular diseases [40,41]. Lipoprotein (a) is a genetically determined parameter and appears to play a central role in the development of coronary artery diseases and thromboembolic events [42,43]. Kamstrup et al. [41] demonstrated, in males with increased lipoprotein (a) levels in combination with other important cardiovascular risk parameters, a 35% higher 10-year risk suffering myocardial infarction. Hartung et al. investigated the aforementioned parameters among OWs. Compared to the group of OWs included in the study by Hartung et al. [39], our study cohort population had a significantly higher rate of elevated lipoprotein (a) levels of >30 mg/dl (36.4% vs. 20.8%). Table 7 shows a comparative survey of cardiovascular risk factors with OWs in other studies.

### 4.2. Metabolic Syndrome

In our study, the definition of the International Diabetes Federation (IDF) was used for the diagnosis of metabolic syndrome [21]. The definition of IDF is well validated and adipositas is the central factor for the definition of metabolic syndrome. In comparison with other studies, the definition of IDF is more likely to be used in central Europe than definitions of the National Cholesterol Education Program (NCEP) and the World Health Organization (WHO) [45,46]. The OWs in our study cohort showed a mean waist circumference of 97.3 ± 11.7 cm. The BMI was within the overweight range with a mean of 26.4 ± 4.1kg/m^2^. With waist circumferences of 84.2 ± 9.0 cm, Japanese OWs displayed smaller abdominal circumferences and normal standard values of BMI [33]. British OWs showed an average BMI that was elevated within the normal range of 24.5 ± 0.9 kg/m^2^ [7]. However, an abnormally large abdominal circumferences and BMI do not appear to be exclusive to our investigated German cohort; they are globally detectable in individuals doing sedentary office work.

One reason for the abnormally large abdominal circumferences in OWs examined in our study seemed to be caused by sedentary work activities, but the impact of this fact is unknown. It is known, that prolonged sedentary activities are associated with larger abdominal circumferences and the severity the associated metabolic risk [47].

According to the criteria of the IDF, the metabolic syndrome was detected in 32.6% of probands in our study. Prior research linked the group of sedentary workers to an unhealthy lifestyle [8]. Bank employees in Russia showed indices in the same range (34.6% prevalence metabolic syndrome) [8]. Compared with office workers from Bangkok, German OWs seemed to dispose of metabolic syndrome more frequently [32,48]. The lowest prevalence of metabolic syndrome was found among office workers in Korea [22]. Table 8 shows a comparison of the prevalence of metabolic syndrome in the professional category of "office workers" among countries. There is increasing evidence in the literature that indicates an increased risk of developing metabolic syndrome following a high rate of sedentary behavior [27].

It can be assumed that differences in professional activities and nutrition exist but are not investigated by prior studies. Therefore, there is a need to perform prospective studies concerning the issue of nutrition. An extensive literature search led to no results for nationwide studies regarding the topic in Germany.

### 4.3. Cardiorespiratory Fitness

High cardiorespiratory fitness is an important factor in the prevention and treatment of cardiovascular risk, diseases, and mortality [49,50]. In detail, higher cardiorespiratory fitness is associated with lower BMI, a lower risk for developing type 2 diabetes, and being physically active [51,52]. Working conditions of office workers are characterized by mainly sedentary working behavior. For a group of OWs, Ramli et al. [53] demonstrated that, for example, being overweight is associated with physical inactivity at work. So far, data about cardiorespiratory fitness of office workers are limited and as far as we know, there are only a few studies about the impact of sedentary working time on cardiorespiratory fitness among office workers. In general, the VO_2max_ values estimated in German OWs seemed to be similar to the range of values in healthy participants (VO_2max_ 2.85 vs. 2.45 L/min^−1^) [54]. VO_2max_ can decline with aging and can differ in reality and self-perception [55,56]. A direct comparison of the data investigated is challenging because of diverging parameters in age and BMI between the study groups. Compared to recreational athletes, the VO_2max_ assessed in our study was, expectedly, lower (VO_2max_, 2.85 vs. 4.7 L/min^−1^) [57]. A group of professional firefighters with high degrees of physical activity at work also showed higher values of oxygen uptake (VO_2max_, 2.85 vs. 3.17 L/min^−1^) [58]. Low VO_2max_ or low exercise capacity is generally linked with higher risk of cardiovascular diseases or higher metabolic risks. [50,59]. A comparison with office workers of other countries showed that the VO_2max_ values of German OWs are relatively high, however, the comparison had some pitfalls due to the lack of availability of study data. Korean OWs showed a relative oxygen uptake of 32.4 ± 5.4 mL/kg^−1^·min^−1^ [60], whereas OWs from Malaysia presented a relative VO_2max_ of 24 ± 3.8 mL/kg^−1^·min^−1^ [53]. In our study, the examined OWs reached a higher mean oxygen uptake of (rel. VO_2max_) 34.1 ± 8.10 mL/kg^−1^·min^−1^. A comparative presentation of relative oxygen uptake is shown in Table 9.

## 5. Conclusions

Sedentary behavior is linked to higher cardiovascular risks [4,63]. To the best of our knowledge, this study was one of the first to assess data on cardiovascular health, metabolic syndrome, and cardiorespiratory status of office workers in Germany. The risk of our German study cohort developing cardiovascular diseases and displaying decreased cardiorespiratory fitness seems to be above the healthy range. In about 50% of the cases, our study cohort showed an increase of diastolic and systolic blood pressure at rest. With respect to international OWs, these values were the highest of the cohorts investigated. The 10-year cardiovascular risk in our study cohort was classified to be less than 5%, assessed by the Framingham risk score. A trend towards obesity could be seen in the investigated individuals doing sedentary office work. However, an abnormally large abdominal circumferences and BMI do not appear to be exclusive to our German OWs cohort; they are globally detectable in the context of "office workers”.

A high prevalence of metabolic syndrome amongst OWs was not exclusive to our German cohort but was identified globally. This highlights that this disease as an international burden, challenging the diagnosis and management for individuals performing sedentary work. Our study aspired to assess the cardiorespiratory status of OWs, but we found a lack of comparable data making no statement possible. As a result of high cardiovascular risk, OWs need to be informed of this risk, and adjustments to the work environment as well as health promoting concepts should be implemented such as corporate sports activities or nutrition courses. To determine the influence of occupational settings in more detail, further national and transnational studies are necessary.

## Figures and Tables

**Table 1:** Basic characteristics of the study population.

	Office Workers			
	*n*	Mean	SD	Median
Age (years)	46	45.8	10.0	48.0
Weight (kg)	46	87.1	13.4	83.8
Height (cm)	46	181.5	5.4	182.0
Body Mass Index BMI (kg/m²)	46	26.4	4.1	25.5
Body-surface area by Mosteller	46	2.09	0.17	2.05
Muscle mass (kg)	46	65.3	6.4	65.7
Body fat (%)	46	20.8	6.5	20.3
Sedentaryf time (h)	46	52.2	17.2	51.5

**Table 2:** Classification of systolic and diastolic blood pressure in degrees of severity [12].

	**Systolic Blood Pressure**						
	***n***	**Optimal (≤120 mmHg)**	**Normal (120–129 mmHg)**	**High Normal (130–139 mmHg)**	**Grade 1 Hypertension (140–159 mmHg)**	**Grade 2 Hypertension (160–179 mmHg)**	**Grade 3 Hypertension (>180 mmHg)**
**Office workers**	46	4 (8.7%)	12 (26.1%)	17 (36.9%)	12 (26.1%)	1 (2.2%)	0 (0%)
	**Diastolic Blood Pressure**						
	***n***	**Optimal (≤80 mmHg)**	**Normal (80–84 mmHg)**	**High Normal (85–89 mmHg)**	**Grade 1 Hypertension (90–99 mmHg)**	**Grade 2 Hypertension (100–109 mmHg)**	**Grade 3 Hypertension (>110 mmHg)**
**Office workers**	46	4 (8.7%)	16 (34.8%)	1 (2.2%)	15 (32.6%)	10 (21.7%)	0 (0%)

**Table 3:** Representation of major cardiovascular risk factors in the study population.

Office Workers						
	*n*	Mean	SD	Median	Normal (%)	Abnormal (%)
Age (Years)	46	45.8	10.0	48.0		
Weight (kg)	46	87.1	13.4	83.8		
BMI (kg/m²)	46	26.4	4.1	25.5	40 (87.0%)	6 (13.0%)
Abdominal waist (cm)	46	97.3	11.7	94.5	19 (41.3%)	27 (58.7%)
Systolic blood pressure (mmHg)	46	129.1	11.7	130.0	17 (37.0%)	29 (63.0%)
Diastolic blood pressure (mmHg)	46	86.6	8.7	90.0	21 (45.7%)	25 (54.3%)
Heart rate (beats/min.)	46	70.1	12.5	68.0		
Tobacco use (Cigarettes/day)	46	1.72	5.53	0.00		
Alcohol (days a week)	46	1.55	1.85	1.00		
Cholesterol (mg/dL)	46	206.6	29.9	204.5		
High-Density Lipoprotein (mg/dL)	46	55.8	14.8	54.5	40 (87.0%)	6 (13.0%)
Low- Density Lipoprotein (mg/dL)	46	118.6	23.6	119.0		
Triglyceride (mg/dL)	46	162.1	91.4	130.0	28 (60.9%)	18 (39.1%)
HbA1c (%)	46	5.4	0.6	5.4		
C-reactive protein (mg/dL)	46	0.27	0.63	0.10		
Grundy et al. [20]						

**Table 4:** Representation of the 10-year risk of cardiovascular events and heart/vascular age of the study population.

	*n*	Mean	SD	Median
**Ten-year risk by Framingham (%)**				
Office workers	46	9.7	9.2	7.4
**Risk categories**				
	**<5%**	**5–10%**	**>10–20%**	**>20%**
*n* Percent	15 (32.6)	13 (28.3)	13 (28.3)	5 (10.7)
**Heart/Vascular Age by Framingham (Years)**				
Office workers	46	49.6	15.5	49.5

**Table 5:** Diagnosis metabolic syndrome number (frequency) according to the criteria of the International Diabetes Federation (IDF) [21].

	*n*	No Metabolic Syndrome	Metabolic Syndrome
Office workers	46	31 (67.4%)	15 (32.6%)

**Table 6:** Spiroergometry and physical activity results of office workers.

	Office Workers			
	*n*	Mean	SD	Median
	**Ventilator aerobic threshold (AT)**			
Hr	46	106.2	19.1	105.0
abs. VO_2_	46	1.47	0.47	1.35
rel. VO_2_	46	18.3	9.4	15.0
%VO_2max_	46	51.0	13.7	49.5
W	46	116.8	42.6	101.0
	**Respiratory compensation point (RCP)**			
Hr	46	129.4	22.0	131.5
abs.VO_2_	46	2.04	0.65	2.05
rel.VO_2_	46	24.0	8.2	24.0
%VO_2max_	46	69.7	16.3	70.0
W	46	171.3	49.0	185.5
	**Maximum Loading (Max)**			
Hr	46	167.7	17.5	167.0
abs.VO_2_	46	2.85	0.52	2.83
rel.VO_2_	46	34.1	8.1	34.5
W	46	257.7	46.9	257.5
W_max/kg_	46	3.03	0.74	3.00
Dynamic METS	46	2212	2292.8	2215

Hr, heart rate (min); abs. VO_2_, absolute maximal oxygen uptake (L/min); rel. VO_2_, relative maximal oxygen uptake(mL/kg^−1^·min^−1^); %VO_2max_, percent of absolute maximal oxygen uptake (L/min).; W, watt; W_max/kg_, watt maximal per kg body weight.

**Table 7:** Comparison of cardiovascular risk factors in published studies of office workers and civil servants.

*n*							
Cardiovascular Risk Factors							
Source	Own Results	Lohsoon-thorn et al. [44]	Suh et al. [22]	Matsuura et al. [33]	Nakanishi et al. [34]	Hartung et al. [39]	Mirmoham-madi et al. [35]
Study Cohort	Office Workers Germany	Office Workers Thailand	Office Workers Korea	Civil Servants Japan	Office Workers Japan	Office Workers Germany	Office Workers Iran
Study cohort	46	531	15109	2335	5275	48	90
Age (Years)	45.8 ± 10.0	42 ± 9.0	41.3 ± 5.9	46.4 ± 10.7	43.5 ± 9.8	30 up to 45	47.5 ± 5.1
Weight (kg)	87.1 ± 13.4						79.8 ± 14.3
BMI (kg/m²)	26.4 ± 4.1	24.5 ± 3.4	23.8 ± 2.6	23.9 ± 3.2	23.4 ± 2.8	25.3	27.5 ± 4.1
Abdominal waist (cm)	97.3 ± 11.7			84.2 ± 9.0			93.6 ± 9.7
Number of smokers (%)	10.9		39.5	43.7		16.7	
RRsRest (mmHg)	129.1 ± 11.7	123.4 ± 14.7	112.8 ± 12.8	124.0 ± 16.0	127.2 ± 14.9		125.2 ± 13.7
RRdRest (mmHg)	86.6 ± 8.7	80.8 ± 10.4	75.7 ± 8.6	77.0 ± 11.0	77.3 ± 11.9		81.3 ± 8.2
Cholesterol (mg/dL)	206.6 ± 29.9				192 ± 32	197	193.4 ± 44.6
HDL (mg/dL)	55.8 ± 14.8	51.7 ± 12.7	50.7 ± 9.9	57.9 ± 14.1	56 ± 13	46	53.3 ± 9.5
LDL (mg/dL)	118.6 ± 23.6			122.6 ± 29.7		135	
Triglyceride (mg/dL)	162.1 ± 91.4	152.2 ± 112.3	127.1 ± 65.9	107.0	108.8 ± 85.1	87.7	169.1 ± 72.9
HbA1c (%)	5.4 ± 0.6					5.2	
CRP (mg/dL)	0.27 ± 0.63					2.3	
Lipoprotein a > 30 mg/dL (%)	36.4					20.8	

**Table 8:** Prevalence of metabolic syndrome in published studies.

Prevalence of Metabolic Syndrome			
Office Workers/Civil Servants			
Source	Prevalence (%)	Country	Criteria
Own results	32.6	Germany	IDF
Lohsoonthorn et al. [32]	25.8	Thailand	Modified NCEP/ATP III
Konradi et al. [8]	34.6	Russia	IDF
Suh et al. [22]	7.4	Korea	Modified NCEP/ATP III
Matsuura et al. [33]	16	Japan	Japanese Criteria

**Table 9:** Published data of relative oxygen uptake (rel. VO_2max_) by office workers and healthy participants.

Office Workers		Healthy Participants	
Source	rel. VO_2max_ (mL/kg^−1^·min^−1^)	Source	rel. VO_2max_ (ml/kg^−1^·min^−1^)
Own results	34.1 ± 8.1		
Ramli et al. [53]	24 ± 3.8	Duque et al. [54]	40.5 ± 5.5
Kennedy et al. [61]	27.1 ± 3.9		
Kraushaar et al. [62]	32.2		
Yoo et al. [60]	32.4 ± 5.4		

## Abbreviations

AT	Ventilator aerobic threshold
BMI	Body mass index
CRP	C- reactive protein
ECG	electrocardiogram
HbA1c%	glycated hemoglobin
HDL	High density lipoprotein
IDF	International Diabetes Federation
LDL	Low density lipoprotein
MET	Metabolic equivalent
OW	Office worker
RCP	Respiratory compensation point
RR	Blood pressure
SBRN	Sedentary Behavior Research Network
rel. VO_2max_	Relative maximal oxygen uptake
abs.VO_2max_	Absolute maximal oxygen uptake
